# Letter to editor regarding “The efficacy and safety of tranexamic acid in high tibial osteotomy: a systematic review and meta-analysis”

**DOI:** 10.1186/s13018-021-02616-x

**Published:** 2021-07-24

**Authors:** Qian Fang, Zhen Zhang, Limin Wang, Wei Xiong, Yunfeng Tang, Guanglin Wang

**Affiliations:** West China Hospital, Sichuan University, Chengdu, 610041 China

Dear editors,

We read a recent published meta-analysis [1] with great interest, which entitled “The efficacy and safety of tranexamic acid in high tibial osteotomy: a systematic review and meta-analysis.” Coincidentally, with a similar analysis we did recently, we have some concerns about reported results and want to share our opinions as well.

Firstly, high heterogeneity was noticed in results of total blood loss (*I*^2^ = 91%, *P* < 0.00001 in *Q* test) between tranexamic acid (TXA) groups and control groups. A similar searching strategy was conducted up to June 7, 2021, and an extra randomized controlled trial [2] was included (Table 1, Fig. 1). We did meta-regression analysis by five items, including total number of patients (*P* = 0.605 > 0.05), age (*P* = 0.052 > 0.05), BMI (*P* = 0.575 > 0.05), preoperative hemoglobin values (*P* = 0.581 > 0.05), and gender (ratio of male/female, *P* = 0.025 < 0.05). Three subgroups were made based on gender: group 1 (> 40%), group 2 (20–40%), and group 3 (< 20%). And random-effects model was performed. Total blood loss was reduced in all three groups significantly: group 1 (*I*^2^ = 57.0%, *P* = 0.127; WMD = − 53.107, 95% CI [− 100.163, − 6.052], *P* = 0.027 < 0.05), group 2 (*I*^2^ = 0%, *P* = 0.751; WMD = − 362.204, 95% CI [− 423.960, − 6.052], *p* = < 0.05), and group 3 (WMD = − 219.471, 95% CI [− 355.615, − 83.328], *p* = < 0.05). Difference between subgroups was also significant (*p* < 0.05) (Fig. 2).

**Table 1:** Characteristics of extra included study

Study	Country	Design	Route	Total samples (M/F)	TXA group				Control group			
					Age (years)	Gender (M/F)	Pre-Hb (g/L)	BMI (kg/m^**2**^)	Age (years)	Gender (M/F)	Pre-Hb (g/L)	BMI (kg/m^**2**^)
Ma 2021 [2]	China	RCT	IV	76 (32/44)	60.78 ± 6.03	14/24	112.12 ± 8.34	24.19 ± 1.98	61.04 ± 5.76	18/20	110.98 ± 8.98	25.05 ± 1.65

*M* male, *F* female, *Y* year, *Pre-Hb* preoperative hemoglobin value

**Fig. 1:**
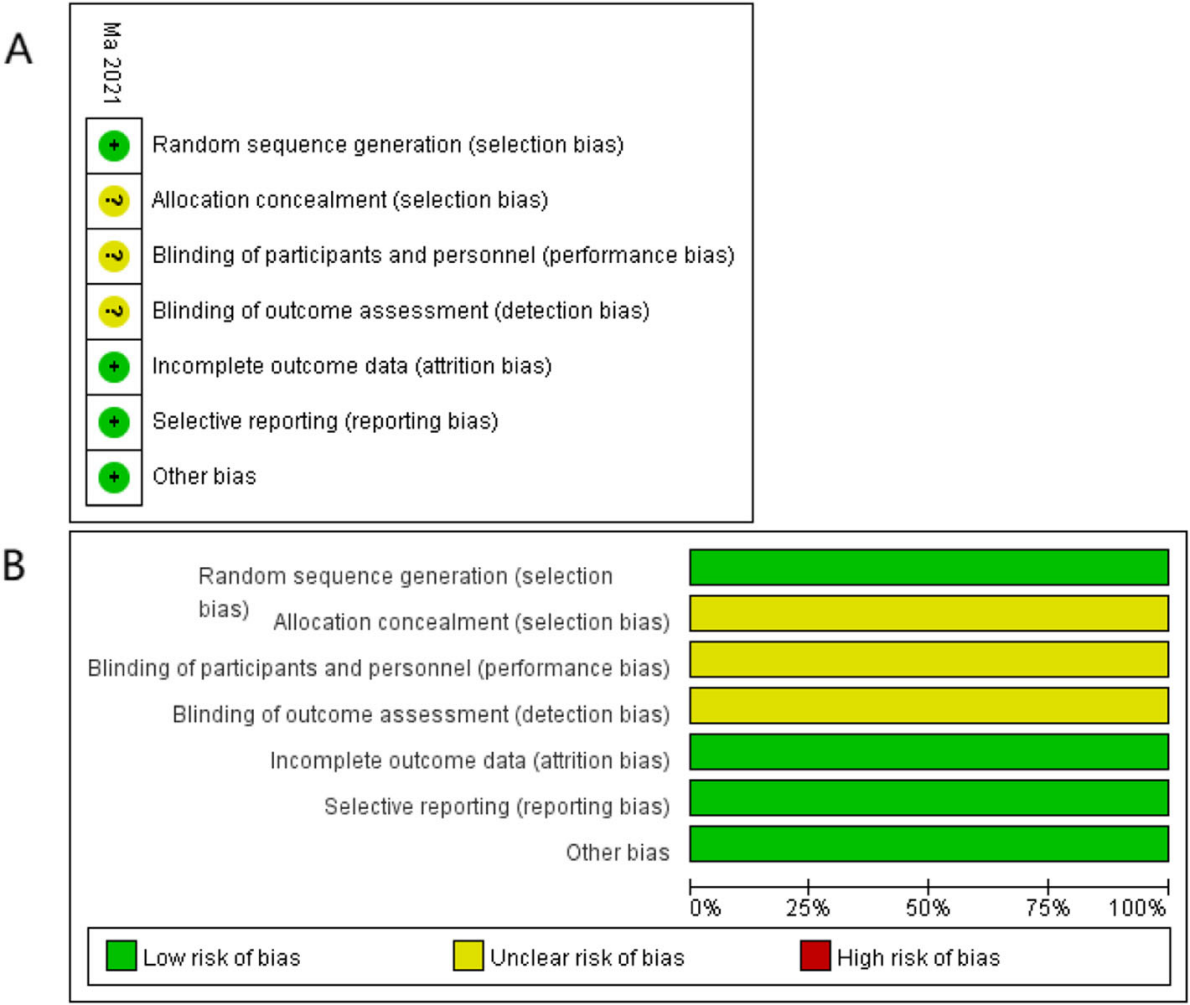
Summary (**A**) and graph (**B**) of quality assessment for extra included study by Cochrane Collaboration’s tool

**Fig. 2:**
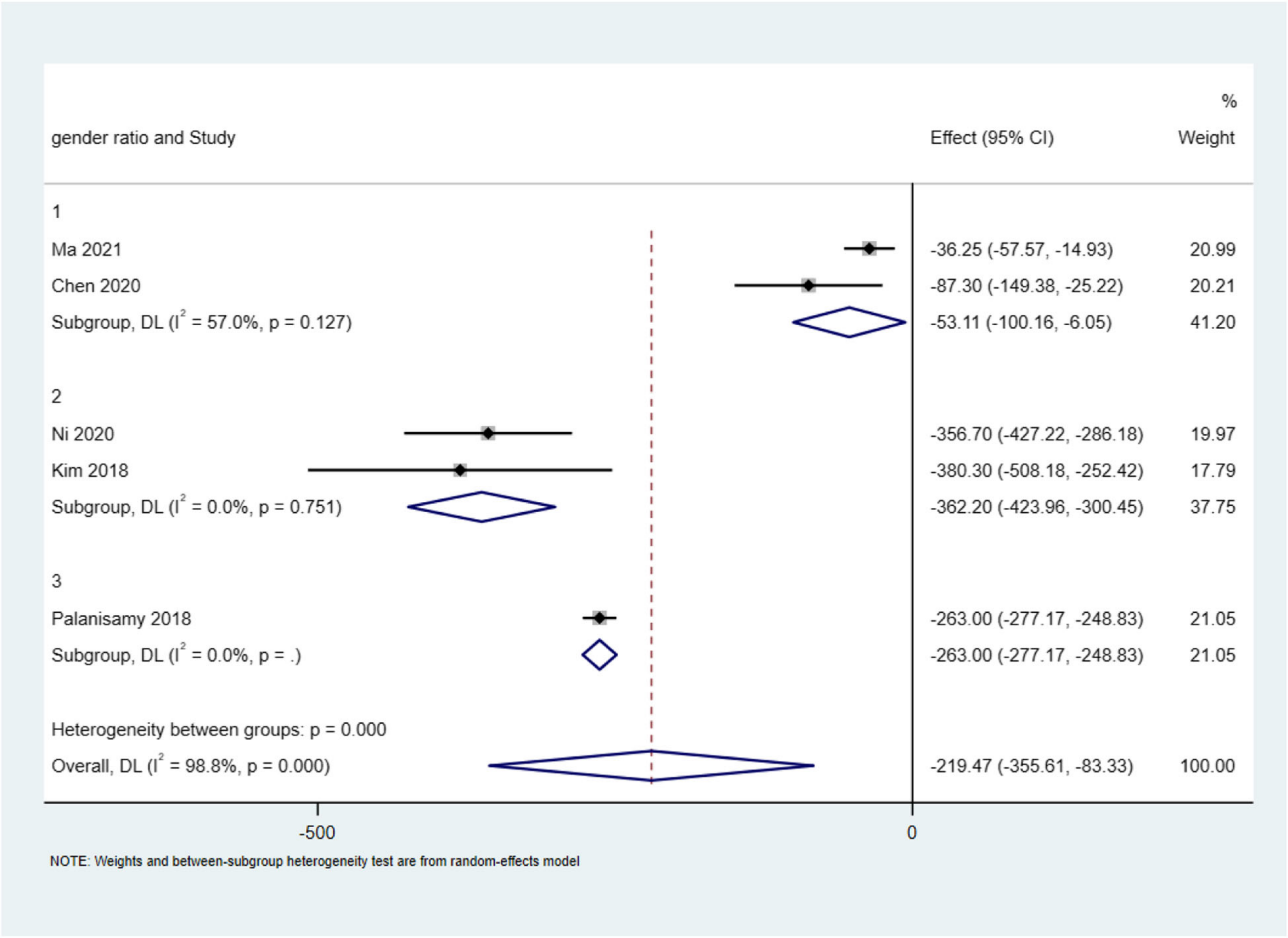
Forest graph of total blood loss in HTO between the TXA groups and control groups by Stata 15.1 software (StataCorp, USA)

The results suggested female might benefit more than male on blood management from TXA. Some studies in arthroplasty replacement [3–5] and orthognathic surgeries [6] presented similar actions. Although the mechanism was still elusive, some hypotheses were given. Sex hormone level was suggested to have effect according to its influence on bleeding levels in hepatic surgery by protecting vascular integrity [7]. Moreover, female might have increased risk of blood loss due to lower preoperative hemoglobin level [8, 9], and TXA had better effect in patients with higher anticipated blood loss [10], which led to the final effect.

Secondly, hemoglobin decrease in the TXA group was significantly lower than that in the control group on post-operation day (POD) 1, POD2, and POD5. Significant difference was also detected in drainage output on POD1 instead of POD2. Because of the short half-time of TXA (about 2 h [11]), we hypothesized intraoperative TXA had a short-time effect, but it might benefit patients for a relatively long time according to significant difference in hemoglobin changing value found on even 5 days after surgery while drainage output only had significant difference on POD1. Other studies also reported the strongest effect of TXA used intraoperatively occurred in the first 24 h after surgery [12].

Finally, analysis of some parameters (hemoglobin decrease on POD2, POD5; drainage output on POD2) were performed with random-effect model, although low risk of heterogeneity was identified (*I*^2^ < 50% and *P* > 0.1). We think fixed-effect model may be better choice. Authors stated it was taken after testing of publication bias; however, we did not find detailed data of it. And trim-filling analysis should be taken with existence of publication bias.

## Data Availability

All data generated or analyzed during this study are included in this published article.
